# Experimental Determination of Coefficients for the Renner Model of the Thermodynamic Equation of State of the Poly(butylene succinate) and Wheat Bran Biocomposites

**DOI:** 10.3390/ma14185293

**Published:** 2021-09-14

**Authors:** Emil Sasimowski, Łukasz Majewski, Tomasz Jachowicz, Michał Sąsiadek

**Affiliations:** 1 Department of Technology and Polymer Processing, Faculty of Mechanical Engineering, Lublin University of Technology, 36 Nadbystrzycka Street, 20-618 Lublin, Poland; e.sasimowski@pollub.pl (E.S.); l.majewski@pollub.pl (Ł.M.); 2 Institute of Mechanical Engineering, Faculty of Mechanical Engineering, University of Zielona Góra, 4 Prof. Szafrana Street, 65-516 Zielona Góra, Poland; M.Sasiadek@iim.uz.zgora.pl

**Keywords:** biocomposite, poly(butylene succinate), wheat bran, thermodynamic equation of state of polymers, p-v-T chart, injection molding

## Abstract

This paper presents the assumptions of a thermodynamic equation of state for polymers according to the Renner model. The experiments involved extruding a biocomposite based on poly(butylene succinate) that was filled with ground wheat bran with its size not exceeding 200 μm. The biocomposite was produced in pellet form with three different contents by weight of wheat bran, i.e., 10%, 30% and 50%. All specimens were examined for their thermodynamic p-v-T characteristics. Taking advantage of the SimFit module of Cadmould 3D-F, experimental results were used to determine the coefficients of thermodynamic equation of state for the tested biocomposite according to the Renner model. The coefficients were then used to calculate transition temperature and to create diagrams illustrating the relationship between pressure, temperature and specific volume for the tested biocomposite. The obtained results can serve as a basis for assessing the suitability of the biocomposite for injection molding, selecting technological parameters of this process, as well as for analyzing shrinkage and defects of injection-molded parts.

## 1. Introduction

Owing to very high costs of launching injection molding-based production, the risk of errors must be minimized at every stage of production process preparation. Thanks to the potential of numerical modeling and computer simulation analysis, many design and technology-related errors can be prevented already at the design stage, which significantly reduces both production costs and production preparation time [1,2,3].

The CAE simulation programs for injection molding use different rheological models for describing the behavior of polymeric materials under pressure at elevated temperature. For this case, the essence of numerical modeling lies in solving equations of mechanics for continuous media (mass, motion, energy). Injection molding is described as a three-dimensional, complex, non-Newtonian and non-isothermal flow; sometimes, it is also described as a non-steady flow. Therefore, the viscosity of polymeric materials predominantly depends on shear rate—it decreases with an increase in shear rate. The viscosity of polymeric materials also depends on temperature and pressure—it decreases with increasing temperature but increases with increasing pressure [3,4,5,6,7].

The thermal state of a polymeric material can be described with the use of physical quantities such as pressure p, specific volume v and temperature T. The relationship between pressure, specific volume and temperature is described by the thermodynamic equation of state, which is also referred to as p-v-T characteristics. Relationships between the parameters of state result from the zeroth law of thermodynamics. Its general form can be expressed by the following equation [8]:

(1) F(p, v, T)=0

The parameters *p*, *v*, and *T* are described as the thermal parameters of the equation of state. To describe the thermal state of a polymeric material, it is enough to know two parameters of this material. In practice, these parameters are usually pressure and temperature, as they can easily be measured nowadays. The p-v-T characteristics describe the relationship between the specific volume v of a given material at the temperature T under the pressure *p* [9,10]. The change in specific volume is also a measure of changes in the density of the material. The rheological characteristics p-v-T are used, among others, for numerical simulation of injection molding, where they are essential for analysis of the pressing phase and mold shrinkage. Moreover, the knowledge of the p-v-T characteristics makes it possible to obtain better results when analyzing the die cavity filling phase. During processing the material changes from the solid to the molten state. Given that the rheological properties of the material in these two states differ significantly, they must be described by separate equations [10,11,12,13].

The most widely used models of p-v-T characteristics for polymers are the Tait model and the Schmidt model. However, neither of these models is continuous at phase transition temperature. Their use for numerical modeling of injection molding may cause problems and produce divergent results, e.g., when modeling pressure changes. It is assumed that both models can be applied with sufficient approximation to both amorphous and partially crystalline materials; yet they are insufficient when it comes to materials containing fillers, which is particularly evident when analyzing mold shrinkage and deformation of injection-molded parts [5,14]. A model that more precisely describes rheological properties of composite materials is the continuous Renner model, which for the solid state is expressed as [8]:

(2) $$v\left(p,T\right)=\alpha_{s}+\xi (\frac{T_{b}}{{\mathrm{CS}}_{5}}+1)\cdot \left(\beta_{M}-\alpha_{s}\right)$$

The equation for the molten state is of the following form [8]:

(3) $$v\left(p,T\right)=\alpha_{M}$$

The constants α_s_, α_M_, β_s_, β_M_ and ξ are described by the following dependencies [8]:

(4) $$\beta_{s}=\frac{{\mathrm{CS}}_{1}}{1+{\mathrm{CS}}_{3}\times p}$$

(5) $$\beta_{M}=\frac{{\mathrm{CM}}_{1}}{1+{\mathrm{CM}}_{3}\times p}$$

(6) $$\alpha_{s}=\beta_{s}+\frac{{\mathrm{CS}}_{2}-{\mathrm{CS}}_{1}}{1+{\mathrm{CS}}_{4}\times p}\times \frac{T_{b}+{\mathrm{CS}}_{5}+10K}{10K}$$

(7) $$\alpha_{M}=\beta_{M}+\frac{{\mathrm{CM}}_{2}-{\mathrm{CM}}_{1}}{1+{\mathrm{CM}}_{4}\times p}\times \frac{T_{b}}{10K};\;\xi =0\;\mathrm{dla}\;x\leq 0;$$

(8) $$\xi ={\mathrm{CS}}_{7}\times x+\left(1-{\mathrm{CS}}_{7}\right)\times x^{{\mathrm{CS}}_{6}}\;\mathrm{dla}\;x>0$$

The transition temperature T_t_ and the reference temperature T_b_ are described by the dependencies:

(9) $$T_{t}={\mathrm{CT}}_{1}+{\mathrm{CT}}_{2}\times p$$

(10) $$T_{b}=T-T_{t}$$

The Renner model coefficients CS_1_, CS_2_, CS_3_, CS_4_, CS_5_, CS_6_, CS_7_, CM_1_, CM_2_, CM_3_, and CM_4_, as well as CT_1_ and CT_2_ are determined analytically based on results of pressure *p*, specific volume *v* and temperature *T*.

The determination of the coefficients of the thermodynamic equation of state for polymers makes it possible to plot the relationship between specific volume *v* and pressure *p* for different temperatures *T*. These diagrams can be used to define injection molding parameters, from the recommended processing temperature through pressure changes in the mold cavity to the duration of the pressing and cooling phases of the molded part, so that the difference in values of specific volume (which is a measure of the expected mold shrinkage) be as small as possible [15,16,17].

The possibility of predicting the behavior of a given material during injection molding, i.e., at variable temperature and pressure, is particularly important when processing new polymeric materials. Given the current ecological concerns, there is a growing interest in polymeric materials that are natural and biodegradable [18,19,20]. One example of such materials is poly(butylene succinate) (PBS), a highly crystalline aliphatic polyester obtained by polycondensation of 1,4-butanediol and succinic acid [21,22,23]. This material has a set of very attractive properties for numerous technological applications, such as very good processing properties that are comparable to those of polyolefins, good thermal and chemical resistance, as well as high impact and tensile strengths [24,25,26]. Melt poly(butylene succinate) is a non-Newtonian liquid that exhibits a decrease of viscosity value effect under the shear forces, which is referred to as the shear thinning behavior [27]. Despite its very good properties, however, PBS is not widely used, which primarily results from its very high price, far higher than that of both traditional polyolefins and other biodegradable materials of natural origin. The high price of PBS is due to a very complicated production process for this material and high costs of substrates [28,29,30]. Expensive polymeric materials can be made cost-effective by adding cellulose and lignin-based natural fillers that are several-fold cheaper and can sometimes even be free waste material [31,32]. PBS is perfect for filling with natural fillers. This is due to its low melting point of 115 °C, which enables a lower processing temperature range and prevents thermal degradation of a filler, as well as due to the hydrophilic nature of macromolecules, which ensures a high degree of material filling, even without the use of a compatibilizer [33,34,35]. The presence of a filler deteriorates the processability of the material by increasing the viscosity of the composition, which, in turn, affects the efficiency of the injection molding process and the drive system load, as well as modifies the crystallization temperature and the degree of crystallinity, thus affecting the cooling behavior of the composition and the characteristics of mold shrinkage [36,37]. In light of the above, it is technologically and economically justified to conduct numerical analyzes of new innovative polymeric compositions in order to determine injection molding parameters that will ensure appropriate flow characteristics and the lowest possible mold shrinkage.

## 2. Experiments

### 2.1. Test Stand

**Figure 1:**
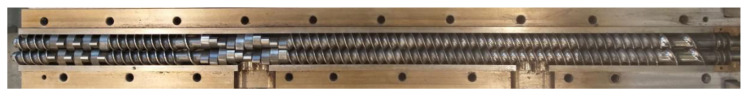
The appearance of an open plasticizing system of an extruder with processing screws.

The poly(butylene succinate)/wheat bran biocomposite was produced using an EHP-2 × 20 Sline co-rotating twin-screw extruder from Zamak Mercator (Skawina, Poland). This extruder has a plasticizing system with nine heating zones. Inside, the plasticizing system has hexagonal core screws provided with replaceable segments, each segment having a diameter of D = 20 mm. The working part length-to-diameter ratio L/D is 40. The employed configuration of the screw segments is shown in Figure 1. The segment system had two kneading zones, the first was described by a double-lobe cam angle of 30°, while the other, located closer to the extrusion head, was described by an angle of 90°. The extrusion head was equipped with a die having two holes with a circular cross section and a diameter of 3 mm. The produced extrudate in the form of two strands was water bath cooled.

The biocomposite strands were then granulated on a G16/32 II granulator from Zamak Mercator (Skawina, Poland). The machine was equipped with a 125 mm diameter rotary cutter with 18 cutting blades. The granulation process was conducted at a rate of 20 m/min with a cutter rotational speed of 320 min^−1^. Granulated biocomposite specimens were used to determine p-v-T characteristics.

A PVT 100 apparatus from SWO Polymertechnik GmbH (Krefeld, Germany) was used to determine rheological properties of poly(butylene succinate) filled with ground wheat bran and to calculate the coefficients allowing for determination of p-v-T characteristics of the material. PVT 100 consists of a measuring system, a hydraulic system, a control and regulation system. The main component of the measuring system is a cylinder in which specimens are placed; the cylinder is closed at both ends by lower and upper pistons. The cylinder allows for generating the maximum pressure of up to 250 MPa and it can be heated to a temperature of 420 °C [38].

### 2.2. Materials

The specimen matrix was made of poly(butylene succinate) (PBS), with the trade name of BioPBS FZ91PB [39], produced by PTT MCC BIOCHEM CO., LTD. This material comes in pellet form and is produced by synthesis of bio-based succinic acid and 1,4-butadienol. PBS is suitable for injection molding of general purpose articles.

The polymeric material under analysis was filled with wheat bran (WB) obtained from a local mill. The pieces of wheat grain husk, bran is a technological waste in the production of white flour and comes in the form of thin flakes with the dimensions of up to a few millimeters. It primarily consists of fibrous substances such as cellulose, lignin and hemicellulose. Other ingredients of wheat bran include phytic acid, oligosaccharides, non-starch polysaccharides, as well as fats and proteins [40]. The preparation process for bran consisted of grinding it twice with a quern, drying it in a laboratory drier for 24 h at 80 °C, and then sieving the dried bran with a moisture content of 3 wt % on a shaker having a column of 5 sieves with decreasing mesh sizes from 1 mm to 0.2 mm, in order to obtain a fraction with a particle size smaller than 0.2 mm.

Prior to extrusion, the appropriate contents of all components were premixed mechanically in a planetary mixer. The obtained loose mixture was then poured directly into the hopper of the twin-screw extruder and subjected to extrusion. During extrusion, the temperature in all heating zones of the plasticizing system of the extruder was maintained at 145 °C. The temperature of the extrusion head was 140 °C. The process was conducted at relatively low processing temperatures (selected via preliminary tests) in order to minimize the risk of thermal decomposition of bran ingredients.

### 2.3. Research Programme and Methodology

The relationship between pressure p, specific volume v and temperature T was determined under isobaric cooling conditions, in compliance with the ISO 17744:2004 standard [41]. Measurements were made with the PVT 100 apparatus from SWO Polymertechnik GmbH (Krefeld, Germany). The test specimens were heated up to the highest tested temperature of 165 °C and pressurized to a preset value (20, 50, 80 and 110 MPa). Next, the pressure was maintained constant and the specimens were cooled down at a rate of 5 °C/min, down to a temperature of 35 °C. The procedure was repeated for a subsequent higher pressure value.

The following were measured with PVT 100: pressure p, specific volume v and material temperature T. A few grams of the material in pellet form were fed into the cylinder of PVT 100. The exact weight of the specimens was weighed with an electronic balance. The specimens of the granulated product were also used to determine density, one of the input parameters necessary for performing analysis with the PVT 100 apparatus [38]. The density of the tested composite depending on the filler content was determined by the pycnometric method.

The results of pressure, specific volume and temperature obtained with the computerized measuring system were initially saved as *.dat binary files. The data obtained with PVT 100 were processed by MS Excel spreadsheet to the *.csv format. The conversion from *.dat to *.csv was necessary for the data to be processed with the use of the specialist SimFit program. SimFit is a feature of the Cadmould 3D-F simulation software. In this study, SimFit V0.6.1 was used. SimFit makes it possible to determine the coefficients of thermodynamic equation of state for polymers and phase transition temperature. The coefficients of thermodynamic equation of state can be determined via one of the two available mathematical models: Schmidt (IKV) or Renner (Simcon) [8]. Given that PBS was filled with wheat bran, the Renner model was selected as the most adequate for approximation.

## 3. Results

Table 1 lists the coefficients of thermodynamic equation of state for polymers written with the use of the continuous Renner model, as determined for pure poly(butylene succinate) and PBS specimens containing 10%, 30% and 50% by weight of ground wheat bran. The coefficients for the equations describing the solid and molten states are written separately. Table 2 gives the transition temperatures of the tested filled specimens and pure poly(butylene succinate), respectively.

The addition of the filler in the form of ground wheat bran did not cause any significant changes in the phase transition temperature of the PBS-based composite. The greatest difference regarding phase transition temperature can be observed for the specimens containing 10% of the filler; a further increase in the wheat bran content caused a gradual reduction in this difference. A similar relationship between the tested samples was obtained in another work of the authors, in which the crystallization temperatures during cooling were determined by means of DSC measurements. The determined crystallization temperature for pure PBS was 78.4 °C, while the crystallization temperatures for the composites were respectively 87.5 °C for 10 wt %, 83.7 °C for 30 wt % and 83.8 °C for 50 wt % of wheat bran [37]. The relationship is analogous to the one presented in Table 2. The bran acts as a crystallization promoter by increasing the transition temperature, but at the same time its high content hinders the formation of crystallites and reduces their size [37,42].

**Figure 2:**
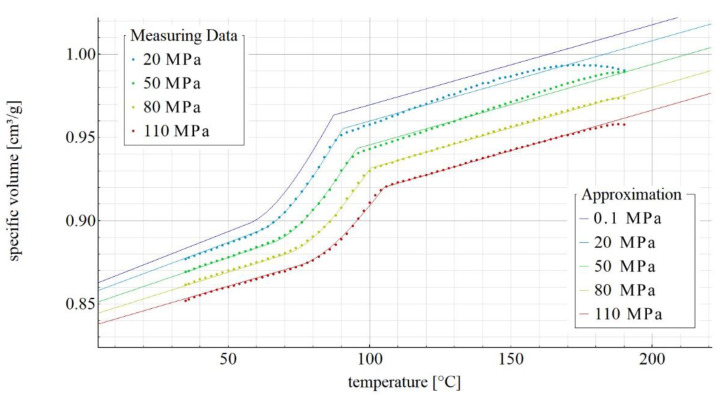
p-v-T diagram for pure poly(butylene succinate).

**Figure 3:**
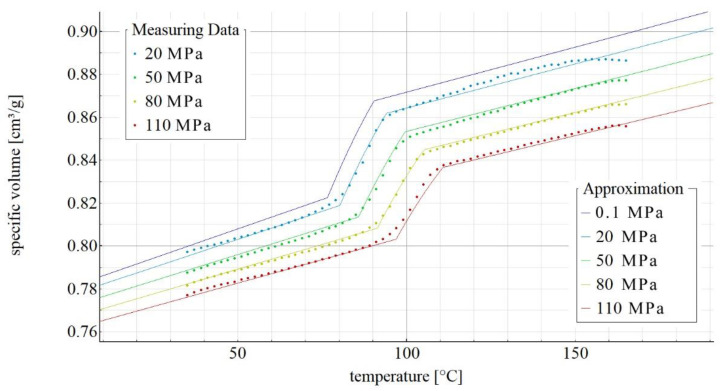
p-v-T diagram for polybutylene succinate containing 10% by weight of ground wheat bran.

**Figure 4:**
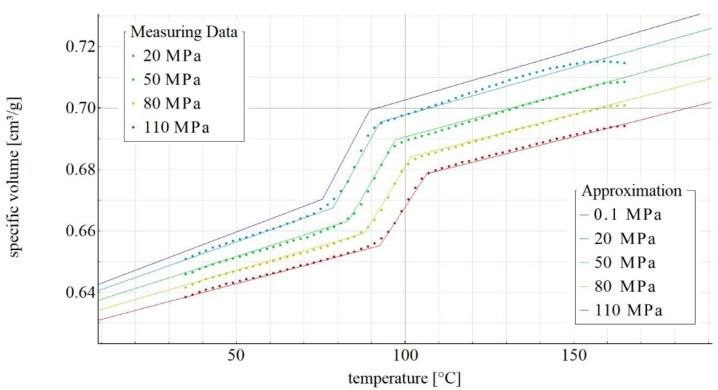
p-v-T diagram for polybutylene succinate containing 30% by weight of ground wheat bran.

**Figure 5:**
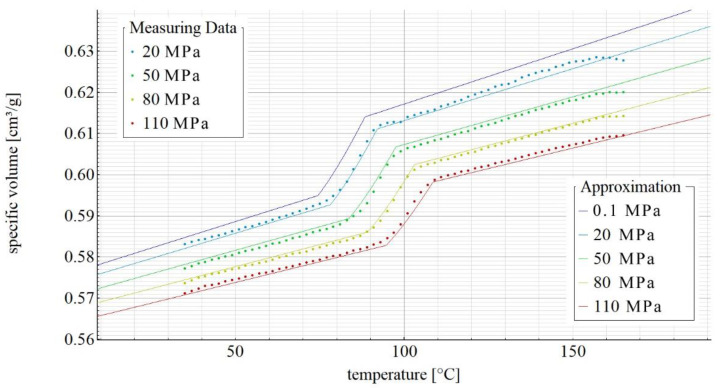
p-v-T diagram for polybutylene succinate containing 50% by weight of ground wheat bran.

Figure 2, Figure 3, Figure 4 and Figure 5 show the p-v-T diagrams illustrating the thermodynamic equation of state for the tested polymer. Figure 2 shows the diagram for the pure poly(butylene succinate), while the diagrams in Figure 3, Figure 4 and Figure 5 refer to the PBS-based biocomposite containing, respectively, 10%, 30% and 50% by weight of the filler. The diagrams were obtained with the use of SimFit. The dots mark the measurement points for individual pressures (20, 50, 80 and 110 MPa) as measured with PVT 100, while the solid lines mark the curves approximated by the Renner equation. As it can be observed, a curve for atmospheric pressure was added to the approximated curves. SimFit makes it possible to add approximated curves to any required pressures, depending on the real process and its needs.

**Figure 6:**
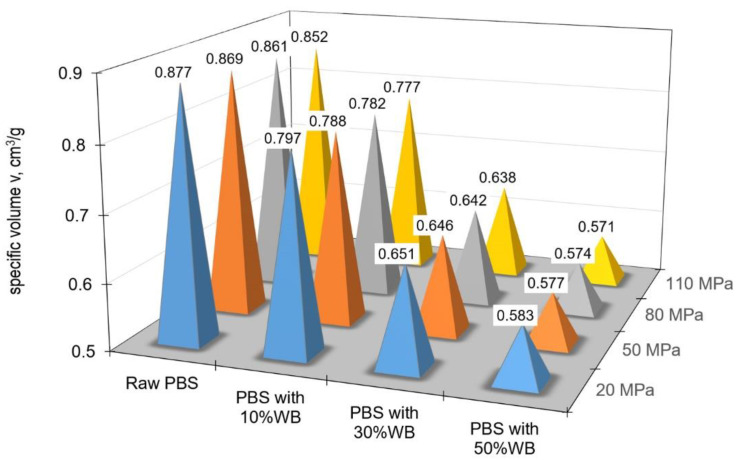
Changes in specific volume depending on pressure and filler content at ambient temperature (35 °C).

**Figure 7:**
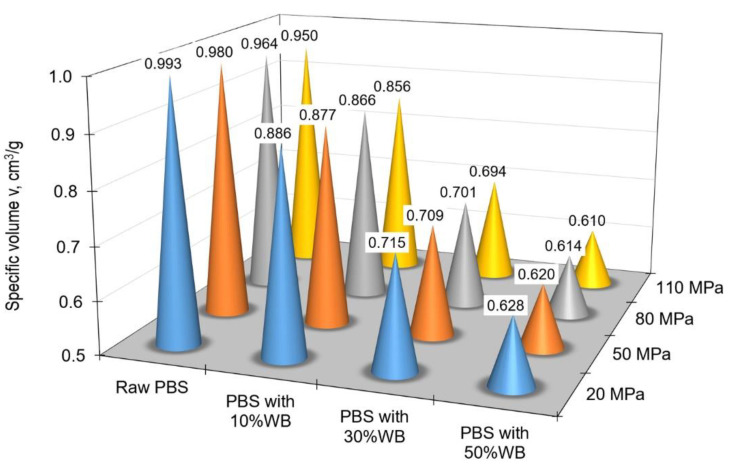
Changes in the specific volume depending on the pressure and filler content at the injection temperature (165 °C).

Figure 6 shows the change in specific volume depending on the pressure and filler content at ambient temperature (35 °C), while Figure 7 illustrates the same relationship for processing temperature (165 °C).

An increase in the filler content led to a decrease in the specific volume v of the obtained polymeric composite. For the lowest tested pressure of 20 MPa, the specific volume of pure PBS was 0.877 cm^3^/g but it decreased to 0.5831 cm^3^/g for the specimens of PBS containing 50% wheat bran. For the maximum tested pressure of 110 MPa, the specific volume decreased from 0.8519 cm^3^/g for PBS without the filler to 0.5712 cm^3^/g for the composite containing 50% of the filler. Intermediate values were obtained for the specimens containing 10% and 30% of the filler. As it can be observed in Figure 6 and Figure 7, the 10% filler content had a small effect on decreasing the specific volume; the specific volume decrease was more significant for the specimens containing 30% of the filler. A further increase in the wheat bran content to 50% had a less powerful effect on decreasing the specific volume of the polymeric biocomposite.

**Figure 8:**
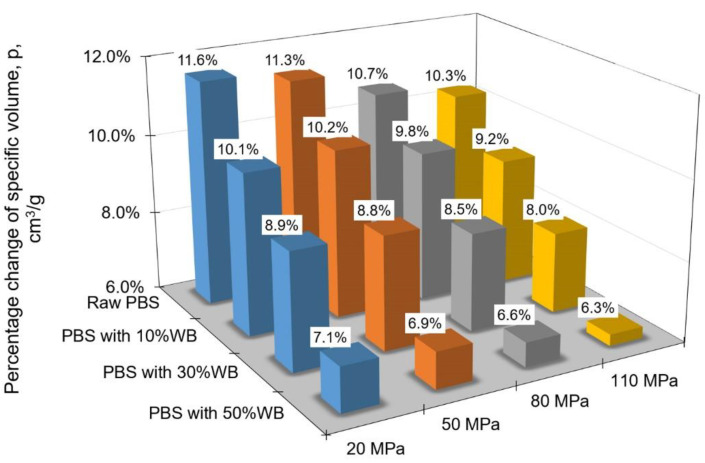
Percentage change in the specific volume of the PBS biocomposite between the processing temperature and the ambient temperature depending on the filler content and injection pressure.

After that, differences between the specific volume at processing temperature and that at ambient temperature were determined and plotted as percentages for individual pressures, depending on the filler content. Obtained results are given in Figure 8.

The data in Figure 8 demonstrate that the smallest difference between the specific volume at processing temperature and that at ambient temperature can be observed for the composite with the highest filler content. For the specimens of PBS + 50% WB it was 7.1% at 20 MPa and 6.3% at 110 MPa. On the other hand, the specific volume of pure PBS changed by 11.6% at 20 MPa and by 10.3% at 110 MPa.

The results obtained with PVT 100 may contain errors due to friction between the melt and the walls of the piston and cylinder, as well as due to potential leaks. Apparatuses that use intermediate fluids (predominantly mercury) for determining p-v-T characteristics are free of such drawbacks; nevertheless, their measurements are also prone to errors due to changes in specific volume of the intermediate fluid, as well as due to the interaction between this fluid and the tested polymer. Differences between results obtained with the use of piston-equipped and fluid-based apparatuses do not exceed 4%.

## 4. Conclusions

The fact that the difference between the specific volume of poly(butylene succinate) at processing temperature and that at ambient temperature decreases with increasing the filler content demonstrates that wheat bran has a positive effect on the mold shrinkage of the tested biodegradable composite.

A proportional decrease in the specific volume of the material with increasing filler content has a positive effect on the behavior of the injection-molded part during cooling, because it results in lower mold shrinkage and greater dimensional accuracy. In addition to that, injection molding can be conducted at lower pressure, which translates into lower energy consumption of processing, the use of smaller machines and less intensive wear of the working components of the injection molding machine. The determination of the specific volume of the material as a function of pressure and temperature based on the coefficients of thermodynamic equation of state for polymers makes it possible to determine the density of this material under processing conditions. In addition, by analyzing mold shrinkage it is possible to determine the shape and dimensions of the mold cavity and pinpoint gates, as well as to determine cooling conditions for the injection-molded part and thus select the appropriate duration of individual phases of the injection molding process.

The obtained coefficients of thermodynamic equation of state for polymers can be used to create a numerical material model in the Cadmould material database featured in the Cadmould 3D-F injection molding simulation software.

## Figures and Tables

**Table 1:** Renner equation coefficients depending on the filler content.

Filler Content, %				
Factor	0	10	30	50
	Solid state			
CS_1_, m^3^/kg	0.00089171081	0.00081710354	0.00066615557	0.00059237701
CS_2_, m^3^/kg	0.00089839098	0.00082257134	0.00067033194	0.00059495441
CS_3_, 1/Pa	2.4512693 × 10^−10^	2.0776941 × 10^−10^	1.9393594 × 10^−10^	1.8095583 × 10^−10^
CS_4_, 1/Pa	3.1531181 × 10^−9^	2.3202444 × 10^−9^	3.938003 × 10^−9^	2.5569665 × 10^−9^
CS_5_, K	30	14	14	14
CS_6_	2	94.79	2	2
CS_7_	0.0054123649	1.0100092	0.78019901	0.58645693
Melt state				
CM_1_, m^3^/kg	0.00096352356	0.00086779105	0.00069931522	0.00061408273
CM_2_, m^3^/kg	0.00096834062	0.00087198181	0.00070251559	0.00061676436
CM_3_, 1/Pa	4.2738994 × 10^−10^	3.3756576 × 10^−10^	2.7754166 × 10^−10^	2.4069352 × 10^−10^
CM_4_, 1/Pa	−1.1940382 × 10^−10^	1.0057286 × 10^−9^	1.4346183 × 10^−9^	3.1890268 × 10^−9^

**Table 2:** Phase change temperatures for pure PSB and composite with variable filler content.

Filler Content	0	10%	30%	50%
Transition temperature CT_1_, K	360.38538	363.61585	362.62081	361.51319
factor CT_2_, K/Pa	1.6391115 × 10^−7^	1.8580051 × 10^−7^	1.5389763 × 10^−7^	1.834749 × 10^−7^

## Data Availability

The data presented in this study are available on request from the corresponding author.

## Abbreviations and Symbols

*p*	pressure
*v*	specific volume
*T*	temperature
CAE	Computer Aided Engineering
CS_1_, CS_2_, CS_3_, CS_4_, CS_5_, CS_6_, CS_7_	(C)oefficients in (S)olid state
CM_1_, CM_2_, CM_3_, CM_4_	(C)oefficients in (M)elt state
CT_1_ and CT_2_	(C)oefficients of (T)emperature
PBS	poly(butylene succinate)
IKV	Institut für Kunststoffverarbeitung, Institute for Plastics Processing located in Aachen, Germany
